# ECZEMA HERPETICUM IN A PATIENT WITH TERMINAL RENAL FAILURE UNDER NARROW-BAND UVB THERAPY

**DOI:** 10.4103/0019-5154.62744

**Published:** 2010

**Authors:** Lorea Bagazgoitia, Sònia Beà, Maite Garate, Ramón Moreno, Pedro Jaén

**Affiliations:** *From the Department of Dermatology, Hospital Ramóny Cajal, Madrid, Universidad de Alcalá de Henares, Spain.*

Sir,

Eczema herpeticum (EH), also known as Kaposi's varicelliform eruption, was first described by Kaposi in 1887. It is characterized by disseminated vesicles and pustules caused by a herpes simplex infection. It most commonly occurs in patients with an underlying dermatosis, especially in atopic patients in whom the epithelial barrier function is decreased. Early onset atopic dermatitis (AD) and high total serum IgE have been identified as risk factors. The relationship between corticosteroid therapy and EH remains unclear. In addition, patients with an inadequate control of their disease (low doses of corticosteroids) may develop EH more easily than those well controlled (higher doses of corticosteroids).[1]

Several other skin diseases such as mycosis fungoides,[2] Darier-White disease,[3] rosacea,[4] tinea cruris[5] and Grover's disease[6] have been related to EH.

**Figure 1:**
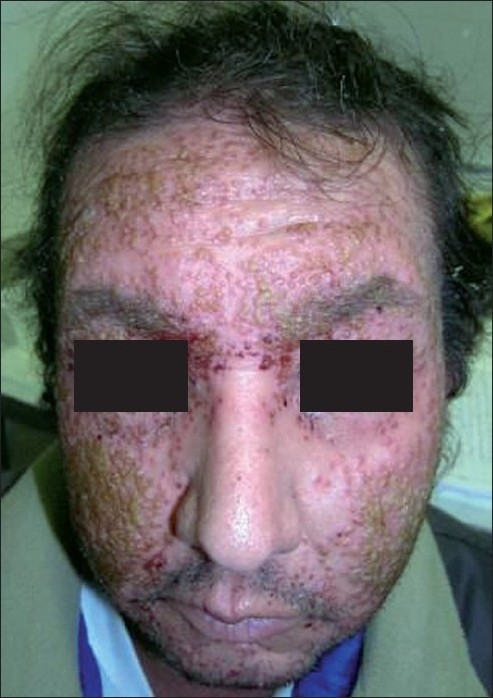
Multiple pustules on the face surface

A 44-year-old man was admitted at our hospital for multiple vesiculopustular lesions localized on the face and trunk which had appeared 2 days before [Figure 1]. The patient had chronic renal failure and had been twice transplanted. He was under immunosuppressant therapy with prednisone 30 mg/24 h and tacrolimus 10 mg/24 h. He referred intense pruritus and dryness of the skin; therefore, he received one session of narrow-band UVB, after which the vesiculous lesions appeared on the face and afterwards spread to the trunk. Tzanck smear showed multinucleated giant cells and acantholytic cells. Therefore, the diagnosis of EH was performed. The patient was treated with intravenous acyclovir (600 mg/12 h), with a noticeable clinical improvement.

Patients with a chronic renal failure have a dry, pale, ichthyotic, scaly skin. These patients usually complain of intense pruritus. Moreover, the reduction of the superficial lipidic layer may predispose these patients to skin infections due to the decrease of the skin mechanical protection, similar to what happens in atopic dermatitis.

In these patients, apart from the renal failure treatment itself, topical treatment for the skin is needed. Emollient and creams containing urea are effective in some patients, but in other cases it is necessary to recur to other treatments such as phototherapy.

To our knowledge, no cases of EH in patients with chronic renal failure have been reported but apparently these patients have predisposition to the spread of herpetic lesions. In our patient the primary herpetic lesion may have been precipitated by the narrow-band UVB, whereas its extension to other body areas might be related to the immunosuppressive therapy. However, similarly to what Wollemberg *et al*.[1] found in AD, the spread of the vesicles might be due to an unsatisfactory control of the skin symptoms and therefore to disruption of the epidermal barrier which could facilitate the extension of the disease.

To summarize, we report the case of an immunosuppressed patient with chronic renal failure, who developed EH after narrow-band UVB therapy. It is therefore advisable, to ask patients with chronic renal failure under immunosuppressant therapy and narrow-band UVB, especially those with a severe skin dryness, about previous herpes simplex infections, in order to make a prophylactic treatment with antiviral medications, such as acyclovir or at least, to take into account the risk of developing EH.
